# The Galenic Heart in the Gothic Cathedral and the Adjournment in Discovery of Circulation

**DOI:** 10.31083/j.rcm2306217

**Published:** 2022-06-16

**Authors:** Laszlo Kiraly, Balazs Gulyas

**Affiliations:** ^1^Paediatric Cardiac Surgery, Department of Cardiac, Thoracic and Vascular Surgery, National University Hospital Singapore, 119228 Singapore, Singapore; ^2^Department of Surgery, Yong Loo Lin School of Medicine, National University of Singapore, 119228 Singapore, Singapore; ^3^Department of Public Health, Semmelweis University, H-1086 Budapest, Hungary; ^4^Centre for Neuroimaging Research at NTU, Lee Kong Chian School of Medicine, Imperial College – Nanyang Technological University, 636921 Singapore, Singapore; ^5^Department of Clinical Neuroscience, Psychiatry Section, Karolinska Institute, S-171 76 Stockholm, Sweden

**Keywords:** history, medicine, heart, anatomy, circulation, Galen, Gothic architecture

## Abstract

**Background:**

Aristotle’s tripartite concept of man—body, soul and spirit—formed the basis of the Galenic system that distinguished nurturing, vitalizing and animating tributary domains, governed by the liver, heart and brain, respectively. The Gothic cathedral structures into similar tripartite arrangements of nave, choir and sanctuary. We studied whether consistent parallels can be found between the Galenic concept of man, the Galenic heart itself and the structuring of the Gothic cathedral.

**Methods:**

Galenic literature along with scholastic texts were reviewed. Examples of Gothic cathedrals were visited and studied in locations. We used medieval analytical tools to compare characteristics of cathedral architecture and contemporary concepts on man and the heart.

**Results:**

Consistent parallels were found between the Galenic system and the structural parts of the Gothic cathedral. The principle of homology, intrinsic to both the Galenic system and Gothic architecture, identified the same tripartite organization in the Galenic heart itself and the segments could be projected onto the cathedral structure. Thus, the physical/nurturing domain was identified with the right ventricle inlet and the nave; the psychological/vitalizing domain corresponded with the right ventricle outlet/interventricular septum and the cathedral’s choir; the animating/spiritual domain paralleled with the left ventricle/aortic valve and the sanctuary in the cathedral.

**Conclusions:**

The Aristotelian/Galenic tripartite concept appears consistent with Gothic architecture and both provided a comprehensive view of the world; their relationship stems in a common philosophical and symbolic foundation. The tripartite interpretation was so coherent that it effectively delayed recognition of circulation and the heart’s role in it.

## 1. Introduction

A passage in *Corpus Hippocraticum * [1] states:

“*The vessels communicate with one another and the blood flows from one to another. I do not know where the commencement is to be found, for in a circle you can find neither commencement nor end, but from the heart the arteries take their origin, and through the vessel, the blood is distributed to all the body, to which it gives warmth and life; they are the sources of human nature and are like rivers that purl through the body and supply the human body with life; the heart and the vessels are perpetually moving, and we may compare the movement of the blood with courses of rivers returning to their sources after a passage through numerous channels.*”

The beautiful text offers a suggestive analogy of blood *circulation*, where the heart is the initiator of flow that is distributed throughout the body [2, 3]. The concept of metabolism is represented as ‘warmth and life’, even the capillaries are anticipated as ‘numerous channels’. *Hippocrates*’ (460–c.375 BCE) possible awareness of the circle of blood flow was forgotten and overtaken by a tripartite system of *Aristotle* (384–322 BCE), *Erasistratus* (c.304–c.250 BCE) and *Galen* (129–c.216) in which the heart assumed a different role [4]. The tripartite concept of man postulated three different (physical, psychological and spiritual) levels of existence [5]. For almost 2000 years, this distributive perception provided a coherent understanding of man [6]. Even when *William Harvey* (1578–1657)—after significant predecessors—described the heart-wise direction of flow in the veins, and the systole as the primary cardiac motion, in *De Motu Cordis* (1628) [7], did not use the term ‘circulation’. He was cognizant of the challenge his observations posed to the pertinent tripartite Galenic system [8].

In this study, we combine atypical subjects and propose a bridge across traditional boundaries among the structures of the Galenic system/heart and the Gothic cathedral. These subjects are quite demarcated in our age, however, it in the age of Gothic—we propose—they were all defined by the same philosophical/theological basis and they all conveyed the same view of the world. The Gothic cathedral re-introduced Platonic-Aristotelian ideas into European architecture [9], and so, it could be interpreted as a tripartite human being [10], body of Christ [11], Heaven on Earth [12]. While the Gothic cathedral represented the tripartite man, it paralleled with the Galenic system and heart, too, by sharing the same philosophical fundament. This foundation was so coherent that it effectively adjourned the discovery of circulation.

## 2. Materials and Methods

Literature on the tripartite Galenic system, medieval scholasticism, related literature of philosophy and theology was reviewed. Gothic church architecture was studied by a focused literature review on its philosophical foundations. Examples of Gothic cathedrals were revisited and studied in locations, primarily, in the South of England. In comparing the structure of the Gothic cathedral and the Galenic system and the Galenic heart itself—representations of medieval thinking—we made an effort to employ medieval analytical approaches. Thus, the *regressus demonstrativus *and the principle of homology, analytical methods of medieval scholasticism were applied [13]. The regressus method consisted of two steps: (1) deduction of reasoned facts from Gothic cathedral architecture and the Galenic system; (2) demonstration of their essential compatibility as well as relative autonomy and the necessity for their conjunction by going back to a deeper root of both—that is, to a tripartite concept. Homologous parallels were drawn with cardiac specimens from a morphological archive in thinking along the Galenic cardiac anatomy.

## 3. Results

### 3.1 The Galenic System: Three Levels of Existence

The tripartite view of creation/division of the world into earth, underworld, and heaven is ubiquitous in human culture. A specific tripartite classification as soma (body), psyche (soul) and pneuma (spirit) was introduced by Aristotle [14, 15]. Erasistratus and *Herophilus *(c.335–c.280 BCE), doctors of the School of Alexandria (3rd century BCE) further developed Aristotle’s order and applied it to human beings [16]. Specificities of the structure and function of the tripartite system were infused into Medieval Europe by the works of Galen [17, 18]. He was an extremely prolific author. Only a fragment of Galen’s output survived, still his works constitute the half of the heritage of ancient Greek texts [19]. Galen remained an undisputed authority until the age of *Vesalius* (1514–1564) [20].

In the tripartite system, each domain has its own function; transports a specific substance *(pneuma) *in ramifying structures and has a governing organ (Table 1).

**Table 1.** **Characteristics of the tripartite Galenic system**.

Domain	Function	Transported substance	Ramifying structure	Governing organ
Physical body (soma)	Nurture, maintain	Pneuma naturalis = venous blood	Systemic veins	Liver
Psychological (psyche)	Vitalize	Pneuma vitalis = vis vitalis = effervescent arterial blood	Systemic arteries	Heart
Spiritual/mental (pneuma)	Animate	Pneuma animalis = animated pneuma, phlegm?	Nerves	Brain

One can read the three domains and their pneumas as different levels of consciousness: vegetative, emotional and mental/moral intelligence, respectively [10]. ‘*Pneuma*’ (πνεῦμα; ancient Greek word for breath, wind, spirit) is a problematic term as it equally signifies the spiritual domain and the substances (‘moving breath’) transported in the respective tributaries [21]. Apart from various technical meanings for medical writers and philosophers of classical antiquity, pneuma is also used in Greek translations of ‘*ruach*’ (רוח) in the Hebrew Bible, and in the Greek New Testament; *see further at 4.2 * [22].

Galen envisaged three distributive and open systems [19]. The liver processes food into blood (*pneuma naturalis*) that is distributed to the organs through the veins. On its passage to the upper body (across the inferior and superior vena cava), venous blood also enters the right ventricle. The Galenic heart only consists of the two ventricles, so the atria belong to the venous system. The right ventricle propels blood backward to the caval system, forwards into the lungs and towards the left ventricle across pores in the interventricular septum. The left ventricle receives aerated mixture from the lungs, as well as the blood across the interventricular pores, that it transforms into vital energy (*pneuma/vis vitalis*) during *diastole*. This combustion-like process also produces heat (ebullition theory) [5]. Effervescent vital energy is then dispensed into the arteries and distributes throughout the organism. The ventricular systole only serves as an exhaust in combustion engines. Pulsation is the intrinsic feature of and generated by the arteries; evoked by the vitalizing pneuma. So, for studying the vital energy of the organism, Galen puts a great emphasis on the observation of pulse quality. The transport is slow in both the venous and arterial systems.

At the top level, the brain is in charge for the animating faculties; it administers *pneuma animalis* via the nerves. Aristotle commented ‘*the brain prevents the heart from overheating*’ [23]. The physical and spiritual domains are connected by the psyche governed by the heart. Aristotelians and Galenists long debated the seat of the soul, where the former argued for the heart and the latter for the brain [24]. Characteristics of the Galenic system in contrast to Harvey’s model are summarized in Table 2.

**Table 2.** **The Galenic system and Harvey’s discoveries**.

Main features of the Galenic system	Harvey’s discoveries
**The heart**:	
Part of the respiratory system; creator of pneuma vitalis	Centre of its own system of arteries and veins; source of circulation
Consists of the two ventricular chambers; atria act as mere hallways (right atrium is part of the liver’s venous/nurturing system); AV valves have no specified roles	4-chambered heart of the atria and two ventricles (confirming Leonardo, Vesalius and Fallopius). All cardiac valves are competent (rejecting Galen)
Pores in the interventricular septum	Interatrial and interventricular septa are intact
The two ventricles contract and relax in one after the other separately and serve different tasks:	Atria and ventricles contract consecutively; bilateral atria and ventricles contract simultaneously (confirming Leonardo without knowing it). The heart is a muscular pump. Cardiac twist: ventricles contract along a spiralling line in space (rejecting Vesalius; confirming Leonardo without knowing it)
∙ The right ventricle receives nourishing blood from the liver and it transmits (1) back to the right atrium, (2) to the lungs (nourishment), (3) through the pores of the interventricular septum to the left ventricle	∙ The atria pump blood into their respective ventricles
	∙ The right ventricle pumps into the pulmonary trunk (confirming Servet); there are no interventricular septal pores (confirming Vesalius in rejecting Galen)
∙ The left ventricle receives warmed and ‘purified’ blood—from the right ventricle through interventricular septal pores—which it pneumatizes with air from the lungs; the left ventricle emits effervescent, pneumatized blood to the aorta along with ‘smokey’ residues	∙ The left ventricle receives blood from the lungs that it pumps out into the aorta
Left ventricular *diastole*—when vitalizing pneuma is formed—is the primary phase of the heart cycle; the purpose of the systole is to exhaust residues	Ventricular *systole* (contraction) is the source of the circulation; ventricles refill during diastole from the atria
Venous blood (produced in the liver) replenishes the peripheral consumption by the organs (relatively small volume). Vitalizing pneuma’s production is the measure of the organism’s well-being	Ventricles eject significant volume of blood per minute (cardiac output)
**The vascular system**:	
Arteries and veins form *two parallel, different* and* open *systems: direction of flow is away from the heart (and liver): centrifugal	Arteries and veins are connected *in series*:
∙ Pneumatized blood (pneuma vitalis) is propelled by the arterial wall’s own oscillation (source of the pulse)	∙ Systemic and pulmonary circuits consisting of arteries and veins are *closed;* arranged in figure-of-eight formation
∙ Nourishing blood (pneuma naturalis) from the liver is distributed through the veins to the organs	∙ Arteries transmit the flow away from the heart. The peripheral arterial pulse is the effect of the left ventricle
∙ There is *central* connection between the two systems in the heart through interventricular septal pores	∙ Direction of the blood flow is *towards the heart* in the veins; venous valves are competent and serve the same purpose (improving on Fabricius)
	∙ Harvey assumes connection in the *periphery* and in the lungs (capillaries were discovered by Malpighi only 1661)
The flow in the arteries is slow; volume of pneuma vitalis unquantifiable	Movement of the blood is fast; the circulating blood volume is significant
**The brain**:	
The heart is under the brain’s control via the recurrent laryngeal nerves and vagal nerves. The brain prevents the heart from overheating (Aristotle)	The heart is autonomous but connects to the brain by nerves

### 3.2 Gothic Architecture

The Gothic style in general and the concept of a cathedral in particular is the product of an organic development in Western European architecture in the 10th–11th century [25]. One may find sufficient examples for precursors of all the technical characteristics of the Gothic including the pointed arch, the ribbed vault and the flying buttress [26]. It is, however, quite unique that we can pinpoint the birth of the style with the start of *Abbot Suger*’s (c.1081–1151) constructions at St. Denis on 14th July 1140 [27]. Abbot Suger’s writings [28, 29] give clear evidence that new liturgical and philosophical concepts came first and then they found its realization in architecture and arts, even in social and economic structures [30]. This new concept was that ‘*man may rise to the contemplation of the divine through the senses*’ [28]. Thus, the cathedral could be perceived as a place where the physical and metaphysical worlds would unite and the worshipper prepares for meeting with God, with the risen Christ in specific [12, 27, 28].

**Fig. 1.**
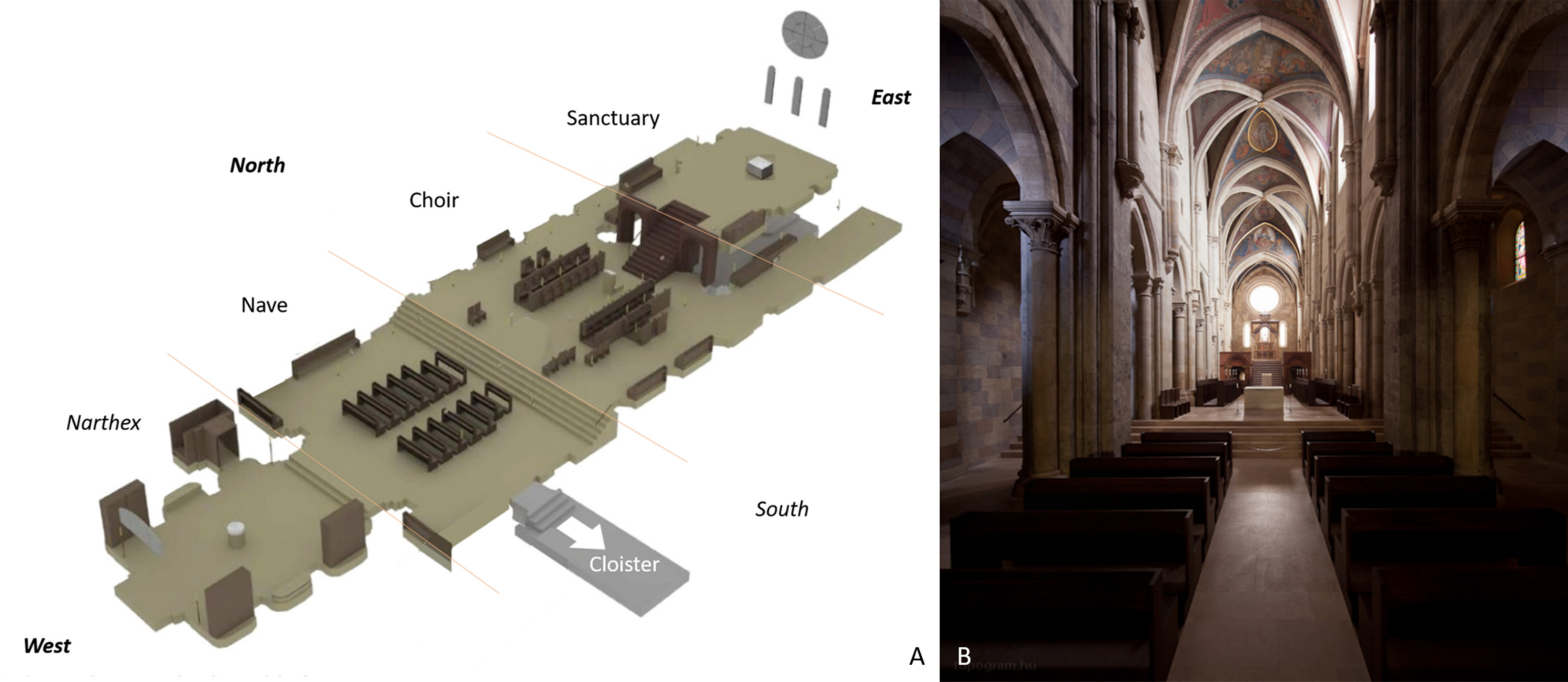
**Gradual elevation of the cathedral floor**. (A) 3D reconstruction of the floorplan of the monastic temple encompasses a smaller nave for the lay community; elevated platform of the expanded choir for the monks; and further elevation of the sanctuary. The narthex is not included in the liturgic space: it serves as a baptistery. (B) West-East view of the same cathedral from the nave towards the sanctuary. Archabbey of Pannonhalma (Pannonhalma, Hungary, early 13th century). Illustrations with permission [32].

Gothic cathedrals structurally and philosophically differ from Romanesque basilicas in separating the longitudinal space into a nave, choir and sanctuary [25]. As for ‘*the mystery had to be protected from the lay community*’ [31], direct view from the Western entrance to the Eastern sanctuary is blocked by by the rood screen (*cancelli*, chancel) and/or a gradual elevation of the segmental platforms (Fig. 1, Ref. [32]). We often find by both gradual elevation of platforms and rood screen stonework e.g., in Canterbury Cathedral (Kent, England, 1070–1834).

The basic floorplan of most Gothic cathedrals forms a cross oriented towards the East in medieval buildings [25, 31]. Identification with the mystical body of Christ is sometimes emphasised by a slight northward deviation of the eastern segment (i.e., the chancel and sanctuary beyond the transcept) to symbolize Christ’s reclining head on the Cross, e.g., in St. Denis (France, 1140–1144), Chichester (West Sussex, England, 1108–1199) and Rochester (Kent, England, 1079–1238) [33]. The primary entrance into the cathedral is through the western façade’s ornate doors that leads to the narthex below the spires. The narthex is typically small (or even absent) and it does not belong to the liturgical space of the cathedral [25]. The nave, the place of the lay community, prepares for the communion. The process is emphasized by the nave’s shear space, glass windows that conceptualize the liturgical context. Thus, the nave belongs to the physical level of existence and further progress is halted by the rood screen (or cancelli) that separates it from the choir (quire). Separation of the profane from the sacred spaces serves the integrity of both [34]. The choir is the middle section of Gothic cathedrals; the place of the clergy, who retired from everyday life and is oriented towards the sacred, and acts as a gatekeeper and mediator. The choir mediates between the physical (nave) and the metaphysical (sanctuary) segments. The sanctuary is the place of mystical ritual that also connects with the metaphysical world by having the tabernacle at its eastern end.

**Fig. 2.**
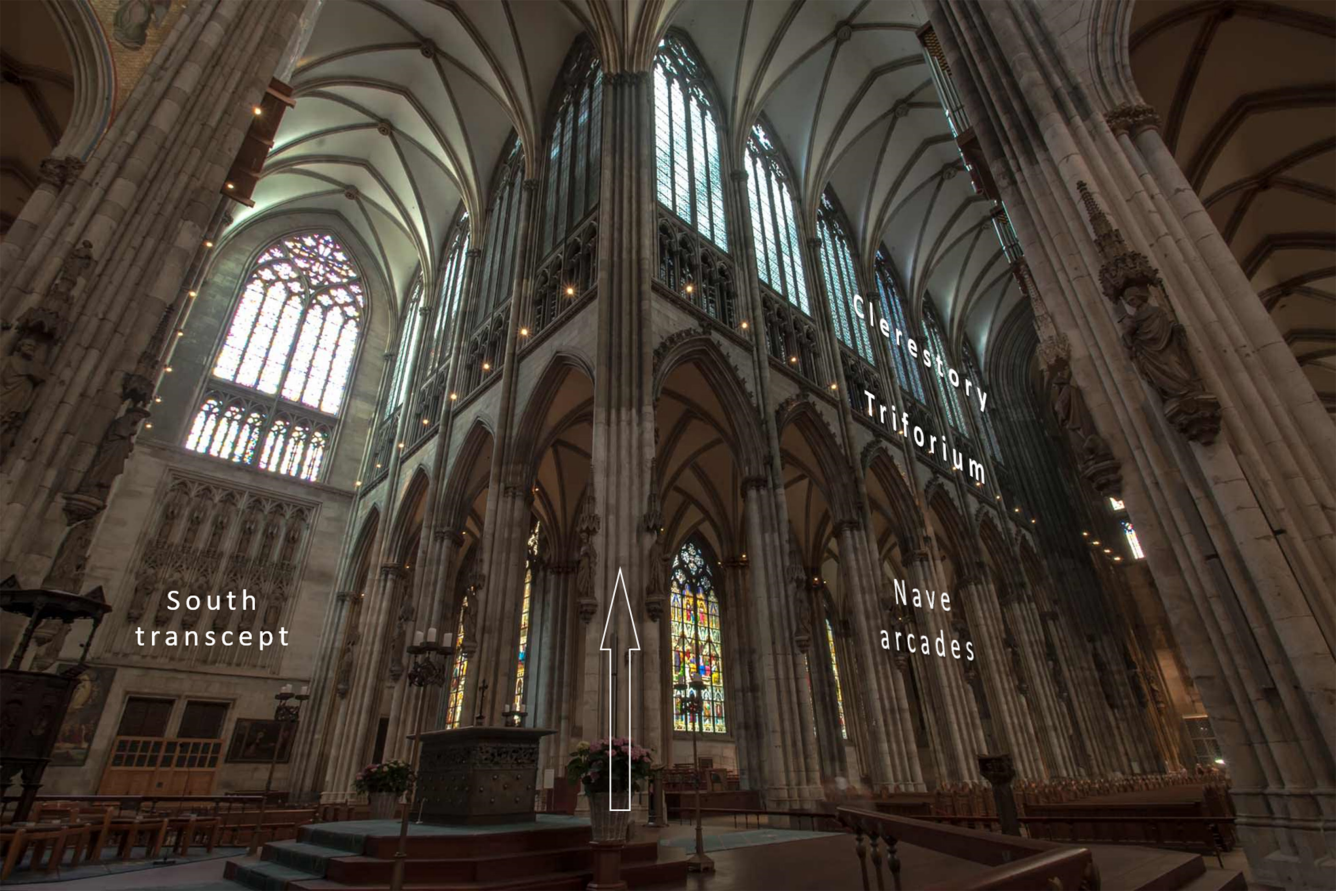
**The vertical structure of the nave repeats trichotomy**. Most Gothic cathedrals display three-storey vertical structure of the nave wall. This consists of a floor’s arcades with the supporting piers; a triforium in the middle that conjoins to the upper level of a stained-glass clerestory. The same arrangement—multiplication of homologous parts—is regularly repeated until nave reaches the transcept. The vertical arrangement restates the Aristotelian tripartite concept. Start of the new horizontal segment is signalled by an uninterrupted pier spanning from the floor to the canopy (*arrow*). Cathedral of Köln, Germany, 1246–1460. Source of photo: Wikimedia Commons [35].

The striking feature of a gothic cathedral is its overwhelming verticality and light. The vertical plane also consists of three grades: (1) the nave’s piers, arcades and flying buttresses (passive entity) that ultimately carry the enormous weight of the canopy; (2) middle level of the triforium (balancing) that conjoins to (3) the enlarged stained-glass clerestory (that becomes an active entity by radiating light) [10]. The vertical structure symbolically repeats the cathedral’s horizontal arrangement (supportive nave, conjoining choir and enlightened sanctuary) (Fig. 2, Ref. [35]).

‘*Bright is the noble edifice that is pervaded by new light*’—writes Abbot Suger [36]. Perception of God as light has numerous previous and contemporary parallels [37], however, light is a new concept in Christian church architecture [23]. Romanesque basilicas stress on the defensive quality of the temple by their thick walls and narrow windows; the seemingly weightless Gothic cathedral permeated by light represents the path that leads from darkness to spiritual enlightment in both horizontal (from West to East) and vertical directions [38]. There are over 60 Gothic cathedrals in the radius of 200 km around London, characterised by the same structural features [39]. English Gothic style puts an emphasis on the longitudinal arrangements, whereas French Gothic is signified by its height. Gothic style also persisted longer in Britain than on the Continent, culminating in the more decorative Perpendicular Gothic style [12]. Furthermore, Gothic cathedrals in England preserve the original segmented structure and furnishing, while the change in the liturgical plan and/or political events resulted in the removal of rood screens, choir stalls, etc. in Continental Europe allowing full perception of space.

## 4. Discussion

### 4.1 Comparison between the Tripartite Concept of Man, the Galenic Heart and the Gothic Cathedral

**Fig. 3.**
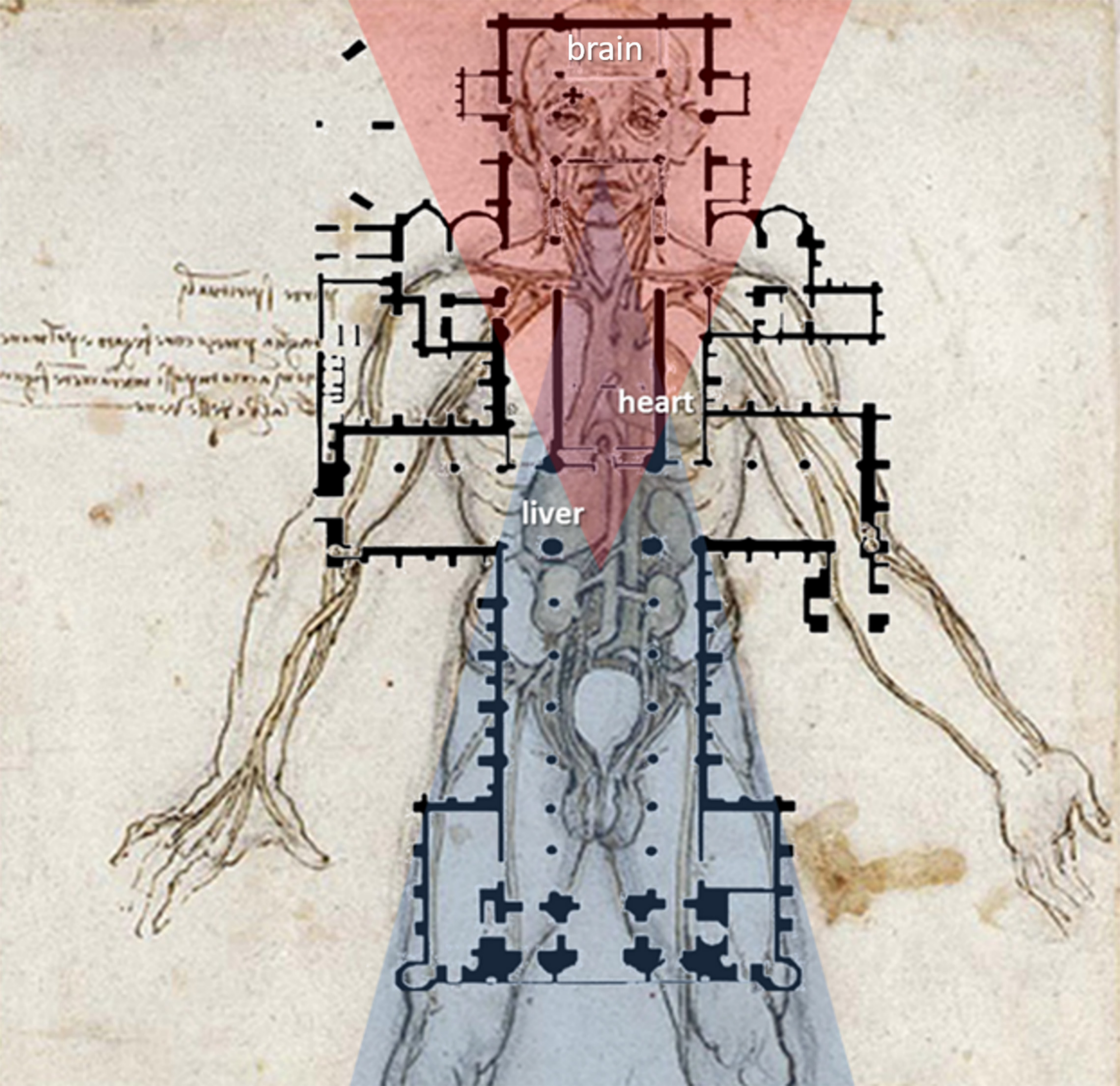
**The cathedral as a human being**. Floorplan of Lincoln cathedral (Lincolnshire, England, 1185–1311) is projected over an anatomic drawing by *Leonardo da Vinci* (The vascular system. 1509; Clark 12597r [46]). Leonardo clearly depicts the Galenic view, where the heart only consists of the ventricles. The liver takes a heart-shape and appears as a pump. It is connected into two major venous tributaries driving upwards and downwards. The liver is situated in the centre of the transcept and the nave of the cathedral. All other viscera under the liver’s rule sit in the nave. This is the level of physical existence. The heart is in the choir that is the domain of the psyche. According to Galen, the brain is the ruler of spiritual faculties. The brain is in the sanctuary of the cathedral. It is apparent that both the cathedral builders and Leonardo—even centuries apart—subscribed the same tripartite classification rooted in the philosophy of Aristotle and Galen. Triangles of metaphysical (red) and physical (blue) domains meet in the middle zone of the heart.

The tripartite Galenic system—based on Aristotle’s and Erasistratus’ philosophical/medical basis—claims three levels of existence that place man in the universe. *Panofsky* proposes that both medieval scholasticism and Gothic architecture is also rooted in Aristotelian and Platonic philosophy [9]. Scholasticism of the 11th–13th century perceived physical and metaphysical worlds in unity [12]. The whole universe would be interpreted as an idealistic existence of measurable harmony that encompasses the physical world [27, 30, 31]. The Gothic cathedral is equally a physical and symbolic representation of that view [12]. ‘*The arrangement of the materials of the church can be likened to the human body. The chancel … represents the head; the cross, from either side, represents the arms or the hands, while the remaining part extending to the west is seen as the rest of the body. The sacrifice of the altar signifies the offerings of the heart… The arrangement of the church signifies a threefold ordering…*’—as *Guglielmo Durando* (1230–1296) states anthropomorphic relations of the cathedral in his *Rationale Divinorum Officiorum* in 1286 [40]. Once the tripartite cathedral is perceived as a living organism, its parallels with the respective Galenic domains become obvious [9, 10, 41]. In this reading, the cathedral’s nave equates with the *soma*, the choir with the *psyche* and the sanctuary with *pneuma*. The nave also represents the physical world ruled by the laws of nature that links it to soma’s main function: maintenance and nutrition. The choir associates with the middle compartment that vitalizes and corresponds with the other two domains. Thus, the heart, the principal organ of the *psyche* resides in the choir of the Gothic cathedral. Other traditions (e.g., Islam) also delegate connecting and communicating quality to the heart [42, 43, 44, 45]. The mental/spiritual faculty governed by the brain animates the organism; and according to scholastic thought, it also connects the individuum to divine metaphysical spheres, especially in the sanctuary of the cathedral [28, 29] (Fig. 3, Ref. [46]).

Gothic architecture—similar to medieval scholasticism—applies methods of ‘*division and multiplication of parts out of parts of homologous parts*’ (*similitudes*) [9]. In medieval thinking, *similitudines* means that a complex entity divided into similar parts should also contain the same organizing principle. By applying this approach, once tripartite parallels are established between the cathedral structure and the Galenic order of man, the same parallels could be found in their homologous building blocks, e.g., in the *Galenic heart* itself. In our interpretation, the Galenic heart features a tripartite organizing principle, therefore it is homologous with the Galenic man and to the cathedral structure, too. Intelligible parallels between one part (i.e., the Galenic heart) and the whole entity (i.e., the Gothic cathedral) corroborate the original hypothesis. The parallel structures have no intrinsic connections but are independent projections of the same organizing medieval philosophical principle.

**Fig. 4.**
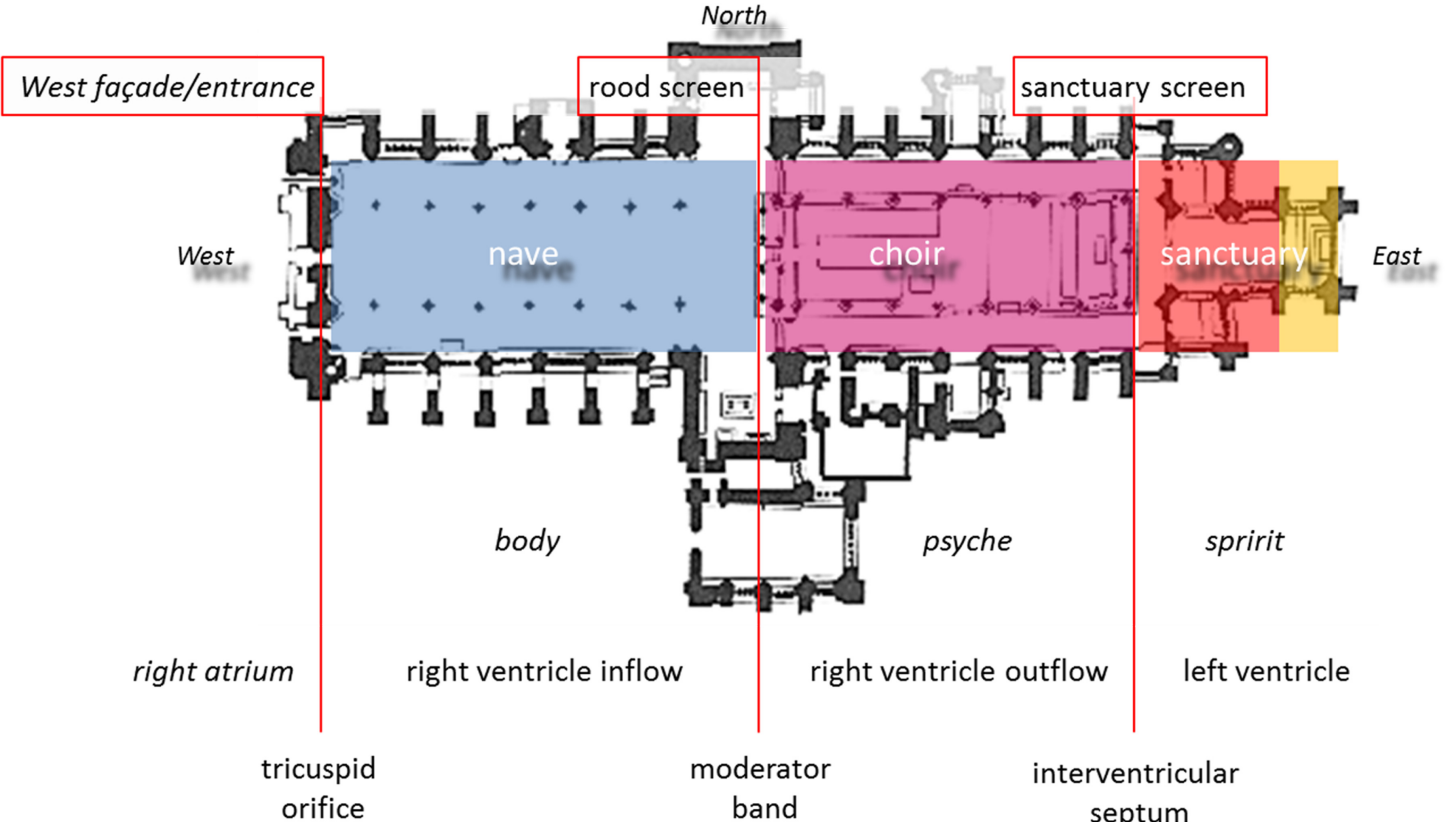
**Segmental structure of the Gothic cathedral in comparison to the different levels of the Aristotelian-Galenic system and the Galenic cardiac segments**. Three segments of the Gothic cathedral: nave (blue), choir (pink), and sanctuary (red-yellow) correspond with the respective Aristotelian-Galenic levels: body, soul and spirit. The yellow area signifies the altar/Eucharist that already belongs to the metaphysical sphere. Architectural segments can be equated to the segments of the Galenic heart. It is of note that in Galen’s view the right atrium was not a part of the heart. Ely Cathedral (Cambridgeshire, England, 1083–1375).

The narthex is not in the liturgical space of the cathedral, nor are the atria part of the Galenic heart. In this understanding, the Western portal represents the tricuspid valve, the nave = the right ventricular inlet; the rood screen = bulbar orifice/moderator band; the choir = the right ventricular outlet, as well as the interventricular septum with Galen’ pores; the sanctuary = the left ventricle (Fig. 4).

**Fig. 5.**
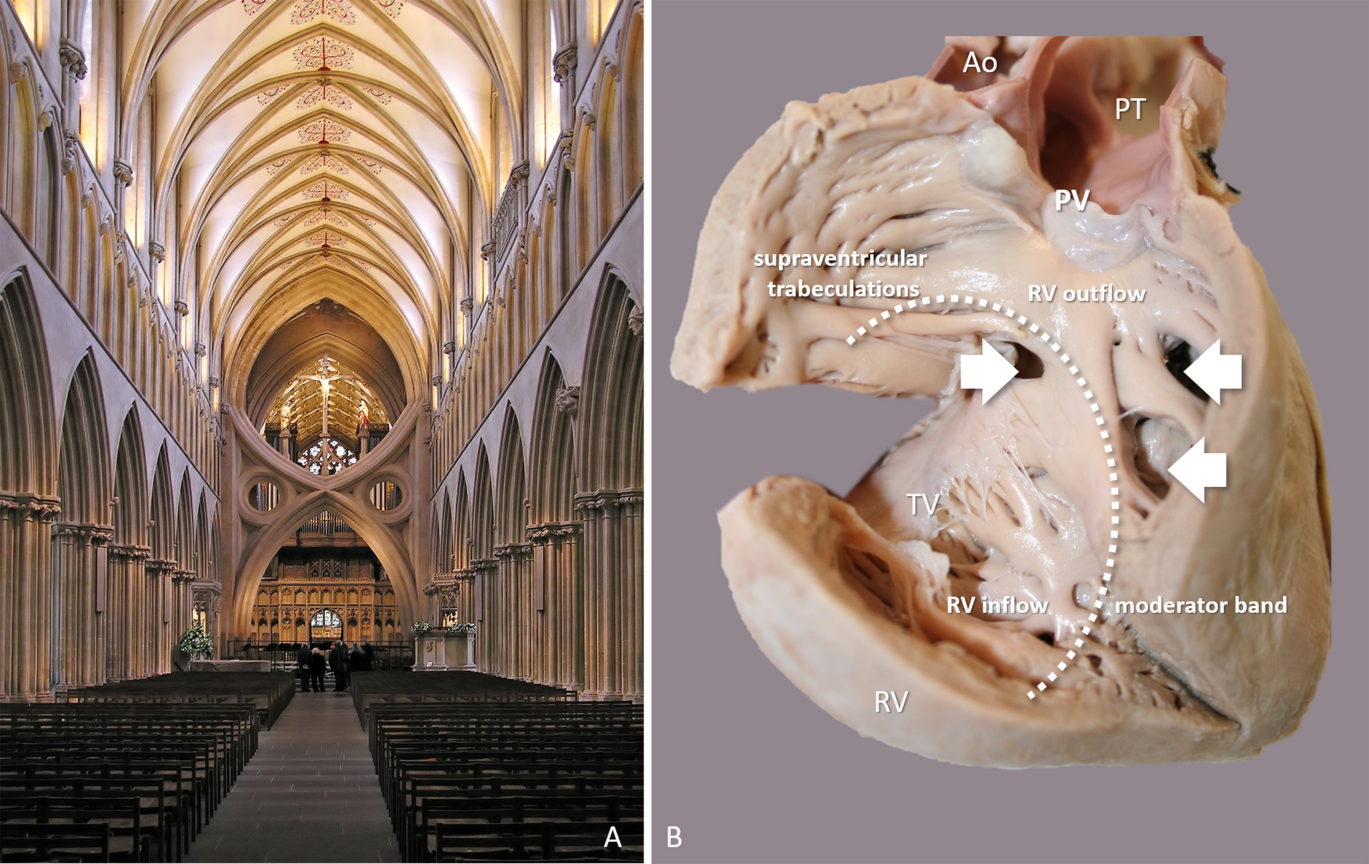
**Separation of the soma and psyche in the Gothic cathedral and in the Galenic heart**. (A) Wells Cathedral (Somerset, England, 1175–1246); view of the nave towards the East. Scissor arches supporting the central tower (1338) stand in front of the rood screen separating the choir. (B) The Galenic heart with the right ventricle free wall opened and lifted: the inflow and the outflow tracts of the right ventricle are separated by the supraventricular trabeculations and the moderator band guarding the bulbar orifice (dotted line). Multiple ventricular septal defects (arrows) appear as Galen’s interventricular pores. The atria (not being parts of a Galenic heart) are not shown in the cardiac specimen. Abbreviations: Ao, aorta; PV, pulmonary valve; PT, pulmonary trunk; RV, right ventricle; TV, tricuspid valve. Author’s photographs.

The Galenic heart is also homologous with the Galenic tripartite man. In this respect, the right ventricle inflow tract represents the soma; the right ventricle outflow and the interventricular septum equates the psyche; and the left ventricle connects with the spirit. The Galenic heart functionally resembles to the liturgical plan of the Gothic cathedral: preparation in the nave equates with the right ventricle inflow that preloads *pneuma naturalis *from the liver. Transition to the next level is simultaneously blocked and facilitated by the rood screen and the bulbar orifice consisting of the supraventricular trabeculations and the moderator band. Meeting with the sacrament is mediated in the middle chamber of the cathedral: the choir; likewise, the right ventricular outflow tract and Galen’s pores mediate the physical to psychological transition. The ritual performed in the sanctuary of the cathedral corresponds to the creation of *pneuma vitalis* that takes place in the left ventricle (Fig. 5).

Aristotle did not consider the atria as parts of the heart, but postulated *three* ventricles [47]. The place and role of the third ventricle remains controversial for it is not clear how Aristotle (and/or his followers) arranged the chambers. Several interpretations have been offered: right ventricle/left ventricle/left atrium [48]; right ventricle/aorta/left ventricle [49]; right ventricle inlet/right ventricle outlet-septum/left ventricle [50]. We adopted the last view on the basis that in the Galenic heart, the right ventricle had distinctive functions: (1) to push unpurified blood back to the caval system either towards the right atrium and the pulmonary trunk, which Galen also considered the part of the venous system; (2) to distill blood across the septal pores towards the left ventricle. So, the Galenic right ventricle inlet and infundibulum had distinct functional differences that reflected in its anatomy. Galen acknowledged the supraventricular crest; however, discovery of the moderator band is historically attributed to *Leonardo da Vinci* (1452–1519) [46]. We acknowledge this interpretation remains short for the role of the left atrium, however, Aristotle and Galen ignored the mitral valve and considered the left-sided chambers as a single unit [49, 50].

**Fig. 6.**
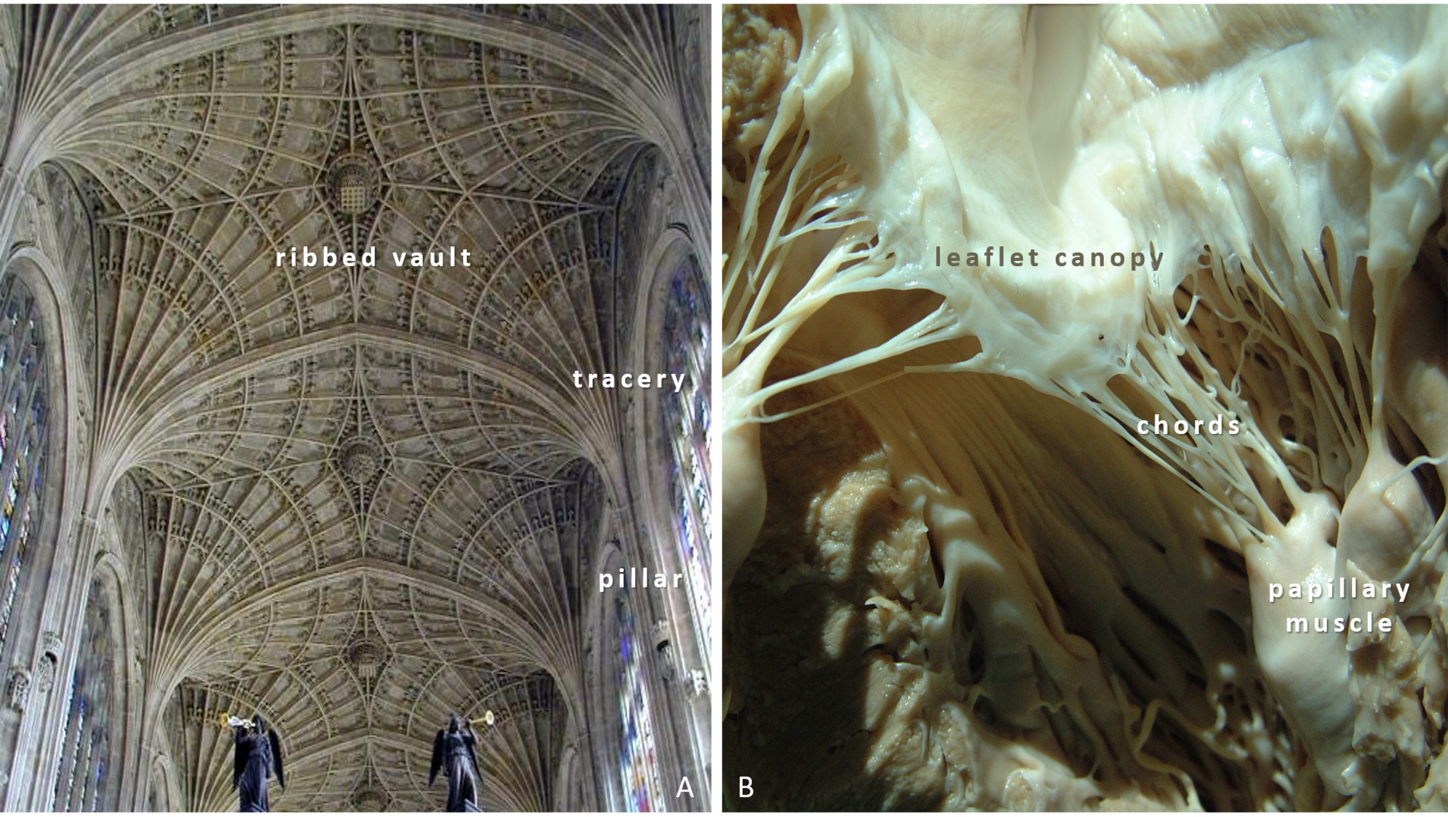
**Closure and opening of corresponding structures**. (A) Perpendicular Gothic ceiling gives the visual effect of having been closed and flattened under pressure. Bath Abbey, Bath, Somerset, England, 1137–1148/1161. (B) Anterior leaflet of the open mitral valve with its chords and papillary muscles corresponding to tracery and pillars. Author’s photographs.

The Gothic cathedral is furnished with rich symbolism—mostly forgotten today—that allows further homologous parallels. According to *Vitruvius *(80/70–c.15 BCE; a Roman architect, highly esteemed by cathedral builders [9, 26, 30]), both the column and pillar are symbols of nature (tree) and of man himself [51]. Pillars, like trees reach to the sky, they root in the ground and their crowns, outpouring out like fans, lead the eyes upwards. In the nave, the scene of events is the Earth, the pillar forest is humanity. The roof simultaneously covers and opens to the metaphysical world as the valve leaflets lock and open. Similarly, the papillary muscles and the cathedral’s pillars support; the chords and the tracery radiate to hold leaflet and canopy (ribbed vault), respectively. These structures, i.e., papillary muscles and pillars are not considered as symbols of each other; they are independent parallels featuring the same organizing principle (Fig. 6).

### 4.3 Symbolic Relationship and Regressus Demonstrativus

Based on phenotypic characteristics, one may indicate *syncretic* relationship between any two entities. Symbols—consisting of present and hidden parts—however, can reveal an immanent relationship between seemingly unrelated articles by grasping on the meaning not on the surface, but at their essence [44]. Platonic thought—adopted by scholastic theologians—postulated *universales*, eternal general ideas that ruled existence and reality; however, eternal ideas (hidden part of a symbol) could not be directly attained by human experience [52]. The perishable physical reality (present part of a symbol) was a mere derivation of the universales*.* Such a symbolic thinking and eschatological view was at the core of medieval man [53]. We conclude the relationship between the Gothic cathedral and the Galenic system and the Galenic heart is coincidental on the phenotypic level but causative on the hidden level.

*Regressus Demonstrativus *is an analytical method attributed to Aristotle (in his *Analytica Posteriora*) that was used by the scholastics to demonstrate that something ‘is’ the case and demonstrating ‘why’ something is the case [13]. Regressus employs inference from an *observed effect* to its *closely related cause* in combination with an inference from the *cause* to the *observed effect.* The *compositive method* provides the cause, and the *resolutive method* knowledge of the effect, respectively. One syllogism is formulated based on an observed effect (i.e., Galenic man and Gothic cathedrals are tripartite structures) and the other on the reasoned cause (i.e., both entities are influenced by Aristotelian philosophy). Table 3 gives an example of the syllogisms.

**Table 3.** **Syllogisms and *regressus demonstrativus* to establish cause/effect relationship between the Gothic architecture and the Galenic system**.

	Observed effect	Reasoned cause	Compositive argument: ‘*a priori*’ proof of the cause	Resolutive argument: ‘*a posteriori*’ proof of the effect
Argument A	Cathedrals that feature tripartite structure are	associated with Aristotelian philosophy.	Cathedrals feature a tripartite structure; hence cathedrals convey Aristotelian thought.	Cathedrals convey Aristotelian ideas; Cathedrals are tripartite structures.
Argument B	The Galenic system that presents a tripartite structure of man is	based on Aristotle’s principles.	The Galenic man is tripartite; hence it presents Aristotle’ principles.	The Galenic system is rooted in Aristotelian philosophy; man is tripartite.
Conclusion	The tripartite structure is a unifying feature that originates from Aristotelian philosophy.			
Regressus demonstrativus	The Galenic system and Gothic cathedrals are related by their tripartite structure on an Aristotelian philosophical basis.			

Thus, with applying the *regressus demonstrativus* to compare the structure/function of medieval entities, i.e., the, we can (1) deduct the necessity for the conjunction of the Gothic cathedral and the Galenic system and the Galenic heart, and (2) demonstrate that their parallels refer to a conjoint philosophical basis, namely the tripartite concept. In other words, they are, indeed, expressions of a common precursor symbolism; they are connected on the hidden level.

### 4.3 Elimination of the Spirit and Soul and Abolition of the Tripartite System

In his later years, *St. Thomas Aquinas* (1225–1274), probably the most important scholastic theologian, realized that faith presupposes and therefore needs natural knowledge of the world [49]. His contemporary, *St. Albertus Magnus* (1200–1280) expressed revolutionary methodological principles: ‘*There can be no philosophy about concrete things*’ and ‘*in such matters only experience can provide certainty*’ [54]. Abbot Suger, builder of the first Gothic cathedral stressed on the importance of human endeavour in the physical world by saying: ‘*The dull mind rises to truth through that which is material*’ [29]. Gothic cathedrals are fine examples for a renewed emphasis on material qualities of human development: ‘*The remarkable power of a unique, single, and supreme reason makes the divine and human natures equal by lessening the disparity between them; and although inferiority of origin and opposition of nature cause the divine and the human natures to appear to be incompatible, a pleasant conformity alone joins them into a single, superior, and measured harmony*’ [29]. Prominence on the material qualities eventually gave rise to critical reasoning, advent of modern science in the Renaissance [55], and the abolition of the tripartite concept. We highlight two critical moments in the latter process.

The 8th Ecumenical Council of the Catholic Church (869–870) in Constantinople amended a doctrine of ‘the two souls’ (Canon XI) [56]. Fighting against Gnostic heterodoxy, church fathers expressed: ‘*though the Old and New Testament teach that a man or woman has one rational and intellectual soul… this holy and universal synod is hastening to uproot this wicked theory now growing like some loathsome form of weed…*’ [57]. Thus, the intellectual soul—*pneuma animalis*, i.e., the spiritual domain in Aristotle’s tripartite system—was effectively abolished and/or sent back to God. Man has become dichotomous, only consisting of body and soul since. Next, the 15th Ecumenical Council of the Catholic Church of Vienne, France (1311–1312) went one step further by defining the human soul (*psyche*) as being essentially and by itself the form of the human body (*soma*) [58]. Seemingly, this was in line with the teaching of Albertus Magnus and Thomas Aquinas based on Aristotelian metaphysics, however, in effect, the doctrine rendered the *psyche* into a derivate of the *soma*. In other words, the Church itself unintentionally opened the doors for materialistic thinking. The scene was set for a new system instead of the tripartite one.

### 4.4 Closing the Circle: The Discovery of Circulation 

In the early 16th century, anatomy was the most popular subject at the European universities. According to contemporary records, there were about 1300 students at University of Padova, *Il Bo*, and all of them wanted to read anatomy [59]. Pupils of Renaissance medicine pursued understanding about the structure of the physical body whereas doctorands of theology sought the seat of soul *in the body*. Expansion of medical knowledge between 1500 and 1550 duly compares to the digital revolution in our age around five hundred years later. New observations challenged Galenic tenets about the heart, the vessels; however, they did not undermine the concept as a whole. Vesalius disproved Galen’s pores in the interventricular septum, established the 4-chambered heart, described opening-closing of the mitral valve; *Fabricius* (1533–1619) discovered the ‘little doors of the veins’, the venous valves; *Colombo* (1510–1559) and *Servetus* (1511–1553) following the insights of the visionary *Ibn al-Nafis* (1213–1288) hypothesized the pulmonary circulation. Despite Ibn al-Nafis had previously accurately described elements of cardiac anatomy and physiology, the majority of his works remained unknown in the West, and—sadly—did not influence discovery of circulation [60]. Nor were the contemporaries aware of Leonardo da Vinci’s anatomical observations and unparalleled illustrations on the heart and the vascular system [13, 61]. Having been alone, scientifically untrained, not knowing Latin, Leonardo freely travelled in an unchartered territory and made discoveries on his own about the 4-chambered heart, interatrial communication, the moderator band in the right ventricle, that the atria and ventricles contract consecutively not simultaneously, the cardiac twist, the closing mechanism of the semilunar valves [62]; the latter two took modern science almost 500 years to prove [63, 64]. Leonardo was surely on the brink of discovery of blood circulation; however, he never trespassed the structure and function divide posed by the Galenic concept [13].

Historical records show that Harvey started his earliest flow experiments under Fabricius in Padua around 1600, and conceptualized his discoveries by 1617–1618 [65]. However, he only published *De Motu Cordis* to pre-empt similar observations by others in 1628 [66]. He might have delayed the publication to mature and refine his concepts [67]. Indeed, he further progressed his theory in the first English language edition of the work in 1653. He might have anticipated scholarly derision, repercussions to his professional status, too [68]. He only discussed motion of the heart *in animals*, direction of flow in the veins, output volumes without mentioning the term, circulation (coined by *Cesalpino* (1524–1603) in 1583) [68]. Harvey assumed a closed circuit but only supposed capillaries (discovered by *Malpighi* (1628–1694) in the 1661) [69]. His discoveries were truly revolutionary despite the lack understanding of metabolism and the role of respiration in it (instead of the then prevailing ebullition theory). There remained an uncertainty of what purpose the new setup would serve. Indeed, Harvey regarded Galen as a distorter of Aristotle’s teaching [68]. In that respect, too, his discovery was ‘revolutionary’—for he revolved around Aristotle’s worldview; as we read in his true Aristotelian dedication to the King: ‘*Most serene King! The animal*’*s heart is the basis of its life, its chief member, the sun of its microcosm; on the heart all its activity depends, from the heart all its liveliness and strength arise. Equally is the king the basis of his kingdoms, the sun of his microcosm, the heart of the state; from him all power arises and all grace stems*’ [7].

We propose that Harvey was not entirely comfortable with the extant consequences of his discoveries, as illustrated by his disputes with *Descartes* and others and the later editions of his work [70]. Like for most of his contemporaries, for him too, Aristotle’s concept provided such a comprehensive understanding about man in universe; a replacement with a mechanistic view raised disturbing questions of the unknown.

### 4.5 Limitations and Refutations

Our work combines atypical subjects and proposes a bridge across traditional boundaries (e.g., architecture, anatomy and philosophy). These subjects are quite distinct in our age, however, it in the age of Gothic they were all defined by the same philosophical/theological basis and they were often practiced by the same masters. We made an effort to avoid projecting 21st century methods of theory of knowledge into medieval subjects. Thus, we applied medieval tools of logic, *regressus demonstrativus* and the principle of homology. We do not imply that parallels between cathedral architecture and the Galenic system/heart were ever drawn by cathedral builders or medieval anatomists. Therefore, all argumentation presented therein is essentially circumstantial as it is not likely to find/have proof of the veracity of the analysis, such as notes from architects that parts of the cathedral were structured on the blueprint of (heart) anatomy as described by Galen. On the other hand, the present perspective of history may allow the discovery of parallels and connotations not obvious to contemporary observers. In the present study, we only applied the principle of homology to draw parallels between the Galenic heart and the cathedral structure. We did not assess the possibilities of a tripartite structure in other principal organs of the Galenic system, like the liver and brain. We are aware of Leonardo assigned a tripartite structure to the brain [8, 46]. Another limitation of the present study that it only focuses on structural characteristics and the four humours, a pertaining concept of physiology and pathophysiology in medieval medicine is disregarded. Connotations with other ‘*naturales*’ of Galenic medicine: elements, temperaments, humours, faculties merit further investigation. Medieval turn-over of knowledge is estimated at a much lesser pace, distribution of new discoveries was not as widespread as it is nowadays. Anatomical definitions and descriptions in medieval medicine were ambiguous in today’s standards making exact identification difficult. Furthermore, contemporary tools of argumentation applied both inductive and deductive logic based on available definitions that weakens their analytical power in 800 years retrospect.

## 5. Conclusions

Aristotle’s tripartite concept of man formed the basis of the Galenic system; it also appeared both in scholastic thought and Gothic architecture and provided a comprehensive view of man and universe. Owing to shared philosophical foundations, it is possible to draw parallels between the Galenic system and the structural parts of the Gothic cathedral. Application of a contemporary analytical tool and the principle of homology—intrinsic to both the Galenic system and Gothic architecture—enables to recognize the same tripartite organization in the Galenic heart and to project it onto the cathedral structure. Correspondence between the Galenic heart and the Gothic cathedral roots in a common background. For many centuries the tripartite concept provided comprehensive view of man in the world and so the discovery of circulation remained effectively adjourned.
